# The Mini-Mental Examination for Children (MMC): Evidence of validity for children with learning difficulties

**DOI:** 10.1590/1980-57642018dn13-040010

**Published:** 2019

**Authors:** Larissa de Souza Salvador, Ricardo Moura, Fernanda Oliveira Ferreira, Peterson Marco Oliveira Andrade, Maria Raquel Santos Carvalho, Vitor Geraldi Haase

**Affiliations:** 1 Graduate Program in Children’s and Adolescents Health, Federal University of Minas Gerais (UFMG), Belo Horizonte, MG, Brazil.; 2 Department of Basic Psychological Processes, Institute of Psychology, University of Brasília, (UnB), Brasília, DF, Brazil.; 3 Department of Basic Sciences of the Federal University of Juiz de Fora, Campus Governador Valadares, MG, Brazil.; 4 Department of Physiotherapy of the Federal University of Juiz de Fora - Campus Governador Valadares, MG, Brazil.; 5 Department of General Biology, Institute of Biological Sciences, Federal University of Minas Gerais, Belo Horizonte, MG, Brazil.; 6 Department of Psychology, Graduate Program in Children’s and Adolescents Health, Graduate Program in Psychology: Cognition and Behavior, Graduate Program in Neuroscience, Federal University of Minas Gerais (UFMG).; 7 National Institute of Science and Technology on Cognition, Behavior and Teaching (INCT-ECCE), Belo Horizonte, MG, Brazil

**Keywords:** neuropsychological tests, learning disorders, intelligence, psychomotor performance, spatial processing, testes neuropsicológicos, distúrbios da aprendizagem, inteligência, desempenho psicomotor, processamento espacial

## Abstract

The Mini-Mental Examination for Children (MMC) is a widely used tool for assessing global cognitive deficits, however,is still unknown whether MMC is sensitive for investigating cognitive profiles associated with learning difficulties (LD).

**Objective:**

Here we investigate the feasibility of using the MMC for screening school-aged children with learning difficulties in spelling and math.

**Methods:**

The MMC and other neurophysiological tests were administered to a sample of 168 children, aged 7 to 12 years. The sample was subdivided into a Control group and LD group (Math Difficulties, Spelling Difficulties, Math and Spelling Difficulties). Diagnostic accuracy was assessed with ROC analysis. Convergent and divergent validity was assessed using correlation analysis.

**Results:**

Performance on the MMC was associated with nonverbal intelligence, age and school achievement. The LD group had significantly lower performance on the MMC than the Control group. Performance on the MMC discriminated LD children with a global accuracy of around 0.80. Associations between the MMC and the other neuropsychological variables were higher for finger gnosis (r=0.40) and generally higher for early elementary school grades. The MMC proved satisfactory for identifying LD children with good accuracy. Nonverbal intelligence, and perceptual/motor abilities play an important role in MMC performance.

**Conclusion:**

The MMC could be a useful instrument for screening children with LD.

## RESUMO

Mini-exame mental para crianças (MMC) é uma ferramenta amplamente utilizada para avaliar déficits cognitivos globais, no entanto, ainda é desconhecido se a MMC é sensível para investigar perfis cognitivos associados a dificuldades de aprendizagem.

**Objetivo:**

Aqui nós investigamos a viabilidade de usar MMC para triagem de crianças em idade escolar com dificuldades de aprendizagem em ortografia e matemática.

**Métodos:**

MMC e outros testes neuropsicológicos foram administrados em uma amostra de 168 crianças de 7 a 12 anos de idade. A amostra foi subdividida em um Grupo Controle e um grupo LD (dificuldade na matemática, na escrita, ou na escrita e na matemática). A acurácia diagnóstica foi analisada através de uma análise de curva ROC. A validade convergente e divergente foi investigada através de análises de correlações.

**Resultados:**

A performance no MMC foi associada com a inteligência não verbal, idade e desempenho escolar. O grupo LD apresentou desempenho significativamente inferior ao Grupo Controle no MMC. A performance no MMC pôde identificar crianças LD com uma acurácia global em torno de 0.80. As associações entre MMC e outras variáveis neuropsicológicas foram maiores para gnosias digitais (r=0.40) e em geral, mais altas nas séries iniciais. O MMC se mostrou satisfatório para identificar crianças LD com uma boa acurácia. A inteligência não verbal, habilidades perceptivas/motoras tem um importante papel na performance no MMC.

**Conclusão:**

O MMC pode ser um instrumento útil para o rastreamento de crianças com LD.

School performance is an important economic asset. Persistent learning difficulties are a main cause of referral for school psychologists and health professionals, as it is associated with negative long-term outcomes, such as low wages and employability and internalizing and externalizing disorders.^1^

Persistent low achievement is associated with risk factors such as socio-economic-cultural deprivation,^2^ parental involvement,^3^ pedagogical inadequacies,^4^ emotional disorders,^5^^,^ 6 intellectual disability,^7^ genetic syndromes,^8^ chronic diseases, such as asthma and diabetes,^9^^,^ 10 sensorimotor impairment and neurological conditions^11^^,^ 12 and specific learning difficulties^13^^,^ 14 etc. Proper management depends on accurate diagnosis. Diagnosis should be informed through detailed clinical and neuropsychological assessments.^15^

Brief cognitive procedures could be useful in the screening of school performance difficulties and ascertainment of referral need. Quick and accurate screening could optimize time and resource allocation in busy school psychological and health practices. Cognitive screening has been used successfully in the case of age-related dementing illnesses.^16^ Instruments such as the Mini-Mental State Examination^17^(MMSE) and the Frontal Assessment Battery (FAB),^18^ have been successfully integrated into geriatric and gerontological practice. Brief cognitive screening instruments have been less successful in the pediatric setting.^19^ Problems with cognitive screening in children relate to the use of poorly standardized measures,^20^ parental report,^19^ unknown correlations with IQ,^21^ requirements on motor dexterity,^22^ literacy requirements,^23^ and lack of developmental sensitivity.^23^

There are numerous neuropsychological batteries for assessing children’s cognitive processes.^24^ These batteries are usually domain-specific, require trained psychologists and long application times. There is a need for simple cognitive assessment screening tasks that will facilitate screening of a range of cognitive domains in a short period.^25^ Such tasks could be integrated into child care routines, assisting in the early detection of cognitive deficits.

The Mini-Mental State Examination (MMSE)^16^ was designed to screen cognitive dysfunctions, assess the severity of impairments, and identify changes over time in elderly individuals with suspected dementing illness. The MMSE is widely used for evaluation of age-related cognitive decline,^17^ but is seldom used for cognitive deficits or developmental delays in children. A child-adapted MMSE version had a short application time (5-7 minutes) across a wide age range (3-14 years).^26^^,^ 27 Understanding of the instructions was independent of socioeconomic status and educational level. Pediatric versions of the MMSE have been used in several countries.^26^^,^ 28 ^-^ 30 A previous investigation of Brazilian children suggests that the Mini-Mental State Examination for Children (MMC), a child version of the MMSE, is useful for rapid assessment of children with cerebral palsy, providing evidence of validity and normative values.^31^ It is still not known whether the MMC can reliably distinguish between typically-achieving children and children referred for learning difficulties in the school context. Establishing MMC accuracy in children with learning difficulties can help improve cognitive assessment by school psychologists and health professionals in schools, primary care, and neurorehabilitation centers.

We investigated the feasibility of using the MMC, a modified version of the MMSE, for screening school-aged children with school achievement problems in Arithmetic, Spelling, or both. Performance on the MMC was also analyzed according to age and sex. 

## METHODS

### Sample

We assessed 168 children (48.8% females) aged 7 to 12 (mean=9.76; SD=1.49) years, from the first to sixth grades in regular public schools. Participants were assessed in two phases. First, we performed a classification assessment using the Arithmetic and Spelling subtests of the Brazilian School Achievement Test (TDE),^32^^,^ 33 and the RavenColored Progressive Matrices (CPM) test.^34^ In the second phase, children were submitted to an individual neuropsychological assessment, described below.

Children performing above the 25^th^ percentile on Spelling and Arithmetic TDE subtests were classified as Controls (*n*=106). Those performing below the 25^th^ percentile on Arithmetic, Spelling and on both subjects were classified as Math Difficulties (MD, *n*=36), Spelling Difficulties (SD, n=13), or Math and Spelling Difficulties (MSD, n= 13), respectively. The group of children comprising these three groups was called Learning Difficulty (LD). Children who had missing data in the tasks were excluded from the sample.

Informed consent was obtained in writing from parents and orally from children. Research procedures conformed to the Helsinki Declaration and were previously approved by the local research ethics board (COEP-UFMG ETIC42/08).

### Assessment tools

Group classification criteria were operationalized using instruments to assess arithmetic and word spelling performance. Nonverbal intelligence, fine motor skills, finger gnosis, visuospatial and visuoconstructional skills, visuospatial and phonological working memory, and word fluency were used as possible convergent and divergent validity indicators for the MMC.^31^ All instruments are described below.

**Mini-Mental State Examination for Children (MMC).** The MMC was adapted for children according to Jain and Passi (2005)^23^(see Moura et al., 2017 for the Brazilian version).^31^ Preliminary versions of the MMC were prepared by two senior researchers with experience in neuropsychological assessment. The final version was decided by consensus. To increase developmental sensitivity, different geometric figures were required for each age level. Choice of age-appropriate geometric figures was based on the Brazilian developmental neurological exam (“Exame Neurológico Evolutivo”).^35^ The adapted version of the MMC comprises 13 items and assesses five cognitive abilities (orientation, attention and working memory, episodic memory, language and constructional praxis) with a maximum score of 37.^31^

**Raven’s Coloured Progressive Matrices (CPM).** Nonverbal intelligence was assessed using the age-appropriate Brazilian validated version of Raven’s CPM.^34^ Analyses were based on *z-*scores calculated from general population parameters provided in the test manual.

**Brazilian School Achievement Test (TDE).** The Brazilian School Achievement Test^32^^,^ 33 is a standardized test. Norms include children from the 1^st^ to 6^th^ grades. It is composed of three subtests: single word Reading, Spelling and Arithmetic. We used the Arithmetic and Spelling subtests. The Arithmetic subtest is composed of three simple orally presented word problems and 45 written arithmetic calculations of increasing complexity. The Spelling subtest consists of dictation of 34 words of varying degrees of frequency, complexity and concreteness, occurring in the final position of orally presented sentences. The TDE has been used in several Brazilian studies of children with learning difficulties, demonstrating both reliability and validity.^36^^,^ 37 Analyses were based on local standards provided by Oliveira-Ferreira et al. (2012).^33^

The domains and instruments below were used in the neuropsychological assessment. Motor dexterity was assessed using the mean times of execution for each hand in the Nine-hole Peg Test (9HPT).^38^ Finger gnosis was assessed using the total accuracy score of the finger localization task.^39^ Visuospatial, visuoconstructional and planning abilities were assessed using the copy of Rey’s complex figure.^40^ The reverse digit span^41^ and Corsi blocks^42^ tests were used to assess the executive component of working memory. Verbal semantic access and association/categorization were assessed using a word fluency test for the categories Animals, Body Parts, Foods, and words beginning with F, A, and S.^40^

### Procedures

All children from first to sixth grades in all the partner schools were invited to join the study. Only children whose parents returned the written consent could participate. Nonverbal intelligence and school achievement assessments were performed in groups of approximately 6 children in the classification phase. The neuropsychological tests were applied in two individual sessions of approximately 30 minutes each. All procedures were conducted in quiet rooms in the children’s schools. Order of application of neuropsychological tests was pseudo-randomized into two different sequences.

### Statistical analyses

All analyses were performed using the SPSS statistical program, considering a 95% level ofstatistical significance.Descriptive statistics were performed to characterize groups regarding age, sex and nonverbal intelligence. One-way ANOVAs were conducted to compare school achievement groupsfor MMC performance. ROC analyses were performed to verify MMC accuracy to discriminate the cognitive performance between controls and children with learning difficulties. Pearson correlations were used to explore the associations between MMC scores and neuropsychological tests. All statistical analyses used age-standardized z-scores in order to control for age differences.

## RESULTS

Results are presented in four main sections: 1) School achievement, age, and nonverbal intelligence; 2) MMC performance according to age, sex and school achievement; 3) Diagnostic accuracy of the MMC; 4) Neuropsychological correlates of the MMC.

### School achievement, age, and nonverbal intelligence

After the classification, 106 children (mean age=9.83; SD=1.41 years) performed above the 25th percentile in both Spelling and Arithmetic tasks, and therefore formed the Control group. The MD group consisted of 36 children (mean age=9.42; SD=1.61 years), and the MSD group was composed of 13 children (mean age=9.38; SD=1.75 years). The SD group consisted of 13 children (mean age=10.54; SD=1.26 years).

There were no significant differences between groups regarding age (F=2.21; df=3; p>0.05) or sex (c²=4.75; df=3, p>0.05). We calculated ANOVAs to investigate differences in nonverbal intelligence among groups. Differences were significant (F=7.09; df=3; *p*<0.002); the post-hoc Bonferroni analysis showed that the Control group had higher nonverbal intelligence than both the MD (*p*<0.002) and MSD (*p*<0.016) groups. The SD group did not differ significantly from other groups, and the MD and MSD groups showed similar scores.

### MMC performance according to age, sex, and school achievement

**Age.** A linear regression analysis was conducted to investigate changes in MMC performance according to age (raw score on MMC was used as dependent variable). MMC scores and age showed a significant, though weak, correlation (*r*=0.33, *p<*0.01). There was a significant association between age and MMC performance (R²=0.10, b=0.052, p<0.001).

Differences regarding MMC performance among age groups were significant (F=5.93; df=5, *p*<0.001). Post-hoc Bonferroni analyses revealed significant performance differences between 7 and 10, 11 and 12 years (*all p*<0.001). No other ages differed significantly.

**Grade.** Global grade differences were significant (F =7.96; df=5 & 162, *p*<0.001). Post-hoc Bonferroni analyses revealed significant differences between grade 1 and grades 4 (*p*<0.001), 5 (*p*<0.02) and 6 (*p*<0.001). Grade 2 differed significantly from both grade 4 (p<0.047) and grade 6 (p<0.004). Grade 3 differed significantly from both grade 4 (p<0.02) and grade 6 (p<0.002). No other grades differed significantly.

**Sex.** There was no significant difference regarding MMC performance (t=1.94; p=0.055) between females (mean=0.14; SD=0.87) and males (mean= 0.15; SD=1.07).

**Figure 1:**
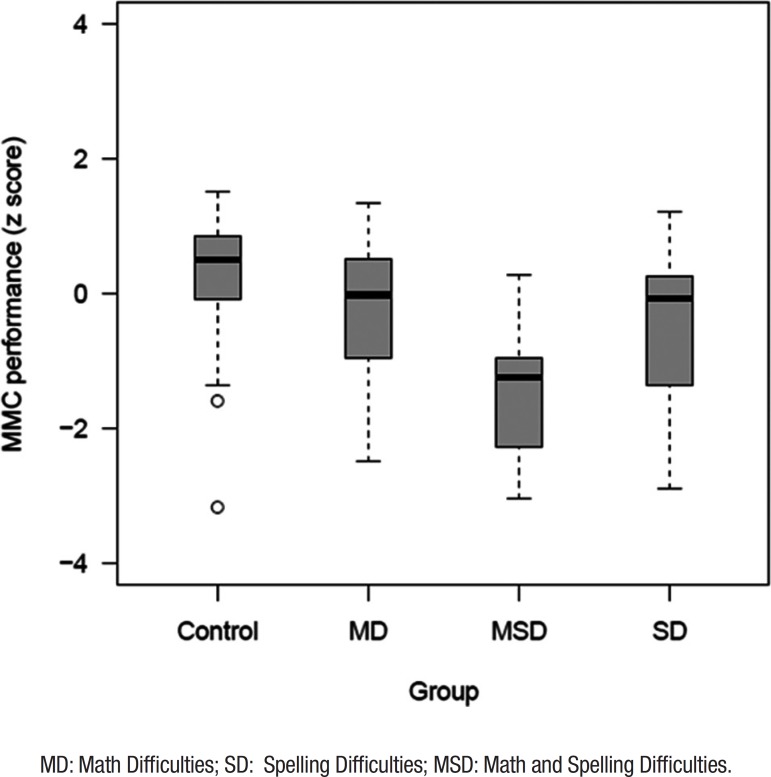
Performance of school achievement groups on MMC. MD: Math Difficulties; SD: Spelling Difficulties; MSD: Math and Spelling Difficulties.

**School achievement.** Figure 1 shows the performance on the MMC of the different groups according to school achievement. Global differences were significant regarding performance on the MMC of the different groups according to school achievement (F=19.28, df=3, *p*<0.001). Post-hoc Bonferroni analyses revealed that the Control group had significantly higher scores on the MMC (mean=35.10; SD=1.60) than all other groups (all *p*<0.05). Comparisons for the MSD group revealed significant differences between this group and the other three groups (all *p*-values<.001), with the MSD group showing worse performance (mean=29.00; SD=3.85). MMC scores for the MD (mean=32.94; SD=3.11) and SD groups (mean=33.69; SD=2.84) did not differ significantly.

### Diagnostic accuracy of the MMC

To verify the validity of the test according to the children’s age range, the sample was further divided into two groups. The Early Elementary School Group (EES) was composed of children from 1^st^, 2^nd^ and 3^rd^ grades (n=68, mean age=8.34; SD=0.89), since these grades showed similar performance on the MMC. The Late Elementary School Group (LES) was formed by children from 4^th^, 5^th^ and 6^th^ grades (n=100, mean age=10.73; SD=0.74), as children from these grades also showed similar performance on the MMC. Table 1 shows sample sizes of groups according to school performance and grade level.

**Table 1:** Distribution of TDE groups according to school groups.

TDE groups	Early elementary school (n=68)	Late elementary school (n=100)
Control	39	67
MD	18	18
MSD	8	5
SD	3	10

MD: Math Difficulties; SD: Spelling Difficulties; MSD: Math and Spelling Difficulties.

**Figure 2:**
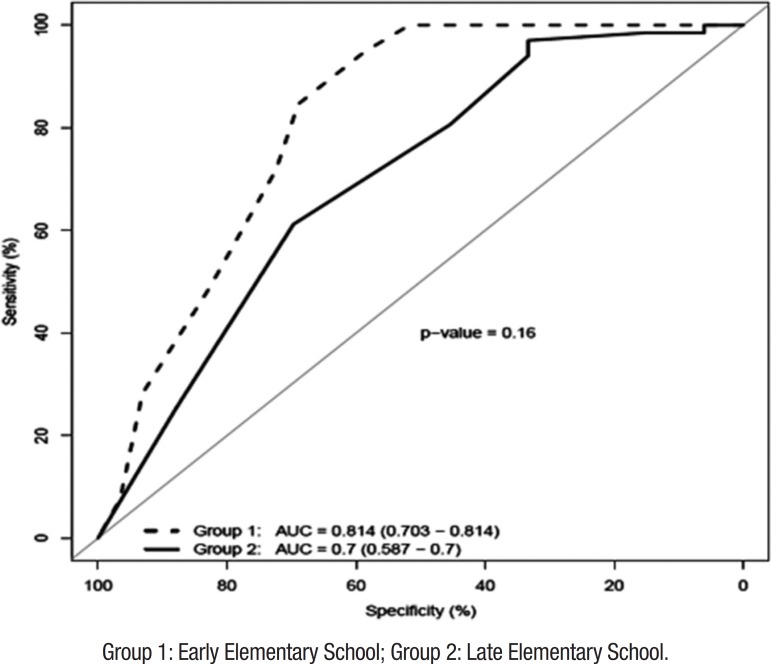
Diagnostic accuracy of the MMC for distinguishing between children with and without learning difficulties. Group 1: Early Elementary School; Group 2: Late Elementary School.

Accuracy of the MMC for distinguishing between typical children and those with Learning Difficulties (LD) was estimated by means of ROC analysis (Figure 2). Results of the diagnostic accuracy were divided into the two School Groups. In the EES, the ROC analysis yielded AUC=0.814, SE=0.057, p<0.001, 95%CI: 0.703-0.95, and in the LES values were AUC=0.700, SE=0.058, p=0.001, 95%CI: 0.586-0.814.

Table 2 presents cumulative percentages of children according to MMC performance. The best threshold of the MMC ROC curve for distinguishing between children with and without school achievement problems was set as a cutoff of 31 in the EES group and 33 in the LES group. These criteria presented a good relationship between sensitivity and specificity for both groups (Table 3). Using these criteria, it was possible to identify approximately 50% of children with LD. The opposite scenario was observed in the Control group, which presented 5% of children below the cut-off in the EES and, 6% of children below the cut-off in the LES.

**Table 2:** Cumulative percentages of children according to the MMC score.

MMC score EES		Cumulative Percentage	MMC score EES		Cumulative Percentage	MMC score LES		Cumulative Percentage	MMC score LES		Cumulative Percentage
Control group	31	5.1	LD group	23	6.9	Control group	29	1.5	LD group	27	3.0
	32	15.4		25	10.3		32	3.0		28	6.1
	33	28.2		27	24.1		33	6.0		30	12.1
	34	51.3		28	34.5		34	19.4		31	15.2
	35	71.8		29	37.9		35	38.8		32	33.3
	36	92.3		30	51.7		36	74.6		34	45.5
	37	100.0		31	58.6		37	100.0		35	69.7
	**N**	**39**		32	69.0		**n**	**67**		36	87.9
				33	72.4					37	100.0
				34	82.8					**n**	**33**
				35	93.1						
				36	96.6						
				37	100.0						
				**n**	**29**						

EES: early elementary school; LES: late elementary school.

**Table 3:** Cut-off scores for the two groups on the MMC according to sensitivity and specificity.

MMC score EES	Sensitivity	Specificity	MMC score LES	Sensitivity	Specificity
22.00	0.000	0.000	26.00	0.000	0.000
24.00	.069	0.000	27.50	.030	0.000
26.00	.103	0.000	28.50	.061	0.000
27.50	.241	0.000	29.50	.061	.015
28.50	.345	0.000	30.50	.121	.015
29.50	.379	0.000	31.50	.152	.015
30.50	.517	0.000	32.50	.333	.030
31.50	.586	.051	33.50	.333	.060
32.50	.690	.154	34.50	.455	.194
33.50	.724	.282	35.50	.697	.388
34.50	.828	.513	36.50	.879	.746
35.50	.931	.718	38.00	1.000	1.000
36.50	.966	.923			
38.00	1.000	1.000			

EES: early elementary school; LES: late elementary school.

### Neuropsychological correlates of MMC

MMC performance was associated with that of all other neuropsychological tasks (see Table 4). Correlations of EES and LES groups are presentedin the upper and lower triangles, respectively. In general, correlations decreased with increase in the grades, ranging from 0.09 to 0.52 for the EES and -0.03 to 0.31 in the LES. No specific pattern of correlates emerged. The highest correlation was between the MMC and the finger gnosis task in both groups. All significant correlations are indicated in Table 4.

**Table 4:** Correlation analysis between MMC and neuropsychological tasks according to school grade.

	(1)	(2)	(3)	(4)	(5)	(6)	(7)	(8)	(9)	(10)	(11)	(12)
(1) MMC total		.44**	-.04	-.03	.27**	.25*	.31**	.17	.25**	.19*	.13	.22*
(2) CPM Raven z score	35**		-.23*	-.09	.23*	.05	.16	.45**	.32**	.30**	-.01	.18
(3) Nine-hole peg test: dominant hand	-.34**	-.48**		.80**	-.01	-.12	-.06	-.11	-.10	-.09	-.15	-.08
(4) Nine- hole PEG TEST nondominant hand	-.36**	-.47**	.70**		-.01	-.04	-.02	-.02	-.01	-.01	-.01	.02
(5) Finger gnosia right hand	.39**	.46**	-.34**	-.34**		.35**	.83**	.15	.08	.06	.12	-.10
(6) Finger gnosia left hand	.19	.47**	-.16	-.25*	.57**		.79**	.01	.18	.04	.1	.07
(7) Finger gnosia Total	.32**	.52**	-.28*	-.33**	.87**	.89**		.08	.12	.07	.14	-.03
(8) Rey Figure Copy	.43**	.21	-.24	-.42**	.30*	.19	.27*		.19	.13	.09	.36**
(9) Digit Span backward	.38**	.43**	-.08	-.25*	.18	.14	.19	.20		.09	.09	.16
(10) Corsi Blocks backward	.21	.27*	-.22	-.39**	.17	.21	.23	.23	.27*		.20*	.11
(11) Verbal word fluency	.31**	.41**	-.29*	-.38**	.33**	.22	.32**	-.04	.41**	.19		.33**
(12) Orthographic word fluency	.24*	.09	-.16	-.22	.32**	.26*	.32**	-.12	.09	-.12	.43**	

* p≤0.05;

** p≤0.01

## DISCUSSION

We investigated the use of the MMC for screening school-aged children with Learning Difficulties (LD). The MMC was applied with other neuropsychological measures in a group of 168 children with and without LD (performance below the 25^th^ percentile in Arithmetic and word Spelling tasks). Our main results were: a) no between-sex differences in performance on the MMC were observed; b) performance on the MMC was associated with nonverbal intelligence, age/grade and Arithmetic and Spelling achievement; c) groups with MD and MSD had significantly lower performance on Raven’s CPM; d) all LD groups had significantly lower performance on the MMC; e) performance on the MMC distinguished LD children with global accuracy of 0.814 in early grades and 0.703 in later grades; f) associations between the MMC and other neuropsychological variables were higher for finger gnosis (r=0.40), and generally higher for early elementary school grades.

In the ensuing sections, we discuss the neuropsychological correlates of the MMC, including general cognitive processing resources and perceptual/motor abilities, as well as its diagnostic accuracy and clinical relevance

### Neuropsychological correlates of the MMC: general cognitive processing resources

One of the most salient results was a correlation of r=0.44 for younger and r=0.35 for older children (r=0.39 for whole sample) between MMC performance and nonverbal intelligence. Children with LD had lower performance on Raven’s CPM in comparison with the Control group (d=0.428). This suggests that some aspects of general nonverbal intelligence are assessed by the MMC. Normal, but low, nonverbal intelligence is a frequent finding in children with learning disabilities such as dyslexia and dyscalculia^43^ and, together with general cognitive resources like executive functions, has been emphasized in recent models of LDs.^44^ Recently, executive deficits in a large sample of teenagers with mathematical learning difficulties were reported.^45^ Concerning learning disabilities, according to Johnson (2012), a single specific or modular deficit, such as phonological processing impairment in dyslexia or numerical processing impairment in dyscalculia, may not be enough to characterize the individual’s performance as impaired. Therefore, general cognitive resources can act as a protective factor for the more specific cases of learning disabilities, like dyslexia and dyscalculia, and also for more heterogeneous multifactorial cases, such as children with LD. When these resources are available, specific deficits or difficulties may be compensated and performance less affected. A disorder may only be characterized when compensatory resources are not available, and the performance impairment overcomes a given threshold. Results are consistent with the importance of general cognitive resources in the impairments observed in LD children, such as the those assessed by the MMC.

### Neuropsychological correlates of the MMC: perceptual and motor abilities

Results also indicated that perceptual and motor impairments present in children with LD may be identified by the MMC. Performance on the MMC was correlated with two important neuropsychological markers: motor dexterity and finger gnosia.

Deficits in perceptual and motor performance are an important correlate of LDs. This has been known for decades, and several developmental neurological exam schedules have been proposed.^46^ IQ is correlated with motor abilities in both typical and atypically developing children.^46^ Perceptual-motor abnormalities are an important predictor of behavioral and school learning difficulties.^12^ Impairments in balance,^47^ handedness,^48^ and body representation^39^ have been observed in dyslexia and dyscalculia. Denckla (2003)^49^ has called attention to the role of perceptual/motor impairments in the diagnosis of LDs. Perceptual and motor impairments may indicate a probable neurological etiology of the difficulties and, at the same time, may co-localize the level of dysfunction in the neuraxis. The MMC may thus be useful to identify accompanying perceptual/motor disorders quickly in children with LDs.

### Diagnostic accuracy: the MMC in clinical practice

Overall, the MMC was abletoidentify LD children with an accuracy of around 80%. A cut-off score of 31 for young children allows the instrument to correctly identify 58% of children with LD, and 5% of children without LD. For older children, a cut-off score of 33 is able to correctly identify around 50% of children with LD, and 6% of children without LD. Our sample size was too small to analyse specific profiles for different LD subtypes (n=13 in the MSD and SD groups). This limitation notwithstanding, results indicated that the MMC could be used in clinical practice as a reasonably sensitive and specific instrument for screeningchildren with LDand normal nonverbal intelligence.The MMC seems to be more sensitive and specific for detecting LD in younger than in older children. Higher clinical value should be ascribed to positive results in younger children than to negative results in older children. It is important to note that the MMC constitutes a screening instrument andis thus not a substitute for comprehensive neuropsychological assessment. It can be of most use in busy clinical practices, identifying red flags indicating referral for more specialized diagnosis and care.

### Limitations and future directions

Despite adding new and relevant insights for the current literature, the findings of the present study have to be seen in light of some limitations. First, we did not control the effect of contextual variables such as socioeconomic status and school setting. Although in Brazil the majority of students in public schools come from low-middle and low socioeconomic classes, there are still some variations in family incomes and educational level that can account for at least a small part of the effects reported here. Furthermore, teaching quality can also vary among public schools.

Besides addressing socioeconomic factors, future studies should also involve larger samples in order to evaluate the accuracy of the MMCfor distinguishing different profiles of learning difficulties, and also using different methodological designs, so that other evidence of validity,such as test-retest, can be provided.
